# Whole-genome sequencing of mutants with increased resistance against the two-peptide bacteriocin plantaricin JK reveals a putative receptor and potential docking site

**DOI:** 10.1371/journal.pone.0185279

**Published:** 2017-09-20

**Authors:** Bie Ekblad, Jon Nissen-Meyer, Tom Kristensen

**Affiliations:** Department of Biosciences, Section for Biochemistry and Molecular Biology, University of Oslo, Oslo, Norway; MRC Laboratory of Molecular Biology, UNITED KINGDOM

## Abstract

By whole-genome sequencing of resistant mutants, a putative receptor for plantaricin JK, a two-peptide bacteriocin produced by some *Lactobacillus plantarum* strains, was identified in *Lactobacillus plantarum* NCFB 965 and *Weissella viridescens* NCFB 1655. The receptors of the two species had 66% identical amino acid sequences and belong to the amino acid-polyamine-organocation (APC) transporter protein family. The resistant mutants contained point mutations in the protein-encoding gene resulting in either premature stop codons, leading to truncated versions of the protein, or single amino acid substitutions. The secondary structure of the *W*. *viridescens* protein was predicted to contain 12 transmembrane (TM) helices, a core structure shared by most members of the APC protein family. The single amino acid substitutions that resulted in resistant strains were located in a confined region of the protein that consists of TM helix 10, which is predicted to be part of an inner membrane pore, and an extracellular loop between TM helix 11 and 12. By use of template-based modeling a 3D structure model of the protein was obtained, which visualizes this mutational hotspot region and further strengthen the hypothesis that it represents a docking site for plantaricin JK.

## Introduction

Bacteriocins produced by lactic acid bacteria (LAB) are antimicrobial peptides that often kill closely related bacteria by inducing membrane leakage [1]. Many of them kill sensitive bacteria by interacting with specific targets, i.e. bacteriocin receptors [2–7] and are considered promising candidates for use as therapeutic drugs [8–11] or as natural additives in food [12, 13]. In order to realize the true potential of bacteriocins, their mechanism of action must be elucidated. In cases where a bacteriocin-receptor interaction exists, revealing the exact bacteriocin docking site is important for understanding the specificity and potency of these peptides. This knowledge is central for rational design of improved peptide-variants.

*Lactobacillus plantarum* is present in a great variety of foods and beverages such as dairy products, vegetables, meat, fish and wine [14]. Selected strains of *L*. *plantarum* are also widely used as probiotics [14, 15]. Several *L*. *plantarum-*strains harbor genes encoding bacteriocins, including so-called two-peptide bacteriocins such as plantaricin JK, plantaricin EF and plantaricin S [14]. These two-peptide bacteriocins consist of two different peptides, both of which are required in approximately equal amounts for optimal antimicrobial activity [16, 17]. Plantaricin JK (consisting of the peptides PlnJ and PlnK) and plantaricin EF (consisting of the peptides PlnE and PlnF) [18] kill sensitive cells by dissipating the electric potential and pH gradient [18]. As for other characterized two-peptide bacteriocins, plantaricin JK kills strains closely related to the bacteriocin-producer, such as *Lactobacillus plantarum* NCFB 965 and the opportunistic human pathogen *Weissella viridescens* NCFB 1655 [16, 19].

By whole-genome sequencing of plantaricin JK-resistant mutants of *W*. *viridescens* NCFB 1655, Oppegård et al. recently identified a member of the amino acid-polyamine-organocation (APC) family of transport proteins as a putative plantaricin JK-receptor [20]. The 610 amino acid long protein is, like other members of the family [21], predicted to contain 12 transmembrane (TM) helices. Mutations that resulted in truncated versions, as well as single amino acid substitutions in the membrane protein, led to increased resistance against plantaricin JK. Point mutations leading to increased resistance were limited to areas either in or near TM helix 10 or in an extracellular loop between TM helix 11 and 12 [20]. The locations of these mutations suggest that this part of the protein interacts with plantaricin JK, either directly (mutations leading to weaker interactions between bacteriocin and docking site) or indirectly (e.g. distortion of 3D structure).

In the present study, the whole-genome sequencing approach was used to identify the plantaricin JK-receptor in *L*. *plantarum* NCFB 965. In addition, we generated two new resistant mutants of *W*. *viridescens* NCFB 1655, as a control and to confirm previous results. An APC protein family member was identified in both *L*. *plantarum* and *W*. *viridescens* as the putative receptor for plantaricin JK, and the structure of a possible bacteriocin docking site in this transporter was investigated using bioinformatics tools for prediction of TM helices and their orientation in the membrane and by template-based modeling of the 3D structure.

## Materials and methods

### Bacteriocin activity assay

The two peptides of plantaricin JK, PlnJ and PlnK, were produced by solid phase synthesis as described by Hauge et al. [22]. The purity of the peptides was at least 50% and the concentrations were estimated by measuring the absorbance at 280 nm, using a molar extinction coefficient calculated from the content of aromatic amino acids. The antimicrobial activity of the bacteriocin against wild type strains and resistant mutants was tested using a 96-well microtiterplate assay system as described by Oppegård et al. [20] in which the concentration of the bacteriocin was added with twofold dilutions in the subsequent wells of each row. The indicator strains used were *W*. *viridescens* NCFB 1655 (LMGT 2314) and *L*. *plantarum* NCFB 965 (LMGT 2003) and were kindly provided by professors I. F. Nes and D. B. Diep at the Laboratory of Microbial Gene Technology (LMG), Norwegian University of Life Sciences. Stationary phase cultures of wild type or mutant variants of either *W*. *viridescens* NCFB 1655 or *L*. *plantarum* NCFB 965 were diluted 1:50 and added to the microtiter plate to obtain a final volume of 200 μl in each well. The microtiter plates were incubated for 6–8 hours at 30°C, after which growth of indicator cells was measured spectrophotometrically at 600 nm. The minimum inhibitory concentration (MIC_50_) value is defined as the concentration of bacteriocin needed to obtain 50% growth inhibition of the sensitive bacteria in the microtiter plate assay. A detailed description of this protocol is publicly available at protocols.io (https://dx.doi.org/10.17504/protocols.io.i66chhe).

### Selection of resistant mutants by treatment with plantaricin JK

Spontaneous mutants with increased plantaricin JK-resistance were isolated as previously described by Oppegård et al. [20]. In short, plantaricin JK was added to a final concentration of 1 μM in the first well of a microtiter plate assay with twofold dilutions as described above. Fresh overnight cultures were diluted 1:100 with MRS medium and incubated for approximately 55 hours at 30°C. Wells with bacterial growth were plated on MRS agar plates. Single colonies were picked and screened for increased resistance against plantaricin JK as described above.

### Identification of genetic changes in mutated strains by whole-genome sequencing

Genomic DNA of mutants derived from *L*. *plantarum* NCFB 965 and *W*. *viridescens* NCFB 1655 was isolated from 6 ml of overnight culture according to the manufacturer’s protocol using the Qiagen DNeasy Blood & Tissue Kit (Qiagen NV, The Netherlands). The genomes were sequenced using an Illumina MiSeq instrument at the Norwegian Sequencing Center and evaluated using the polymorphism detection tool VAAL [23] as previously described by Oppegård et al. [20]. In the evaluation, we used a previously described genome assembly of *W*. *viridescens* consisting of 243 contigs [20]. A crude assembly of the *L*. *plantarum* genome consisting of 188 contigs was obtained from wild type sequence reads using the A5-miseq assembly pipeline [24]. Larger insertions not detected by VAAL were identified by aligning the Illumina reads to the wild type genome assembly using Geneious (Geneious v. 7 created by Biomatters, accessible from http://www.geneious.com). The genome assemblies are available at the European Nucleotide Archive (ENA) with the following accession numbers: PRJEB22014 for the strain *L*. *plantarum* NCFB 965 and PRJEB10916 for the strain *W*. *viridescens* NCFB 1655.

### Template-based 3D structure modeling

A 3D structure model of the APC family protein from *W*. *viridescens* NCFB 1655 was built using I-TASSER (https://zhanglab.ccmb.med.umich.edu/I-TASSER/), an automated web-service for protein structure and function predictions based on amino acid sequences as input. In addition to the structure evaluation report performed by I-TASSER, an evaluation of the backbone RMSD values was done using MOLMOL (V1.0.7) [25]. The highest ranked model built by I-TASSER was used to investigate the mutational hotspot region in the plantaricin JK-receptor. The PDB-file of Model 1 was submitted to the Protein Model DataBase (https://bioinformatics.cineca.it/PMDB/main.php) with the following PM-id: PM0081188.

## Results and discussion

### Mutant strains with increased resistance towards plantaricin JK

The wild type *L*. *plantarum* NCFB 965 strain is about 400 times more sensitive to plantaricin JK than the wild type *W*. *viridescens* NCFB 1655 strain (Table 1). Four *L*. *plantarum* and two *W*. *viridescens* colonies with increased resistance against plantaricin JK were collected. The *L*. *plantarum* mutants had obtained full resistance (over 100,000 fold), whereas only partial resistance (about 50 fold) was obtained with the *W*. *viridescens* mutants (Table 1). For the wild type strains, the MIC_50_ values of plantaricin JK were measured to be 0.01 nM for *L*. *plantarum* and 4 nM for *W*. *viridescens*.

**Table 1:** Mutants of *L*. *plantarum* NCFB 965 and *W*. *viridescens* NCFB 1655 with increased resistance against plantaricin JK.

Strains	MIC_50_ value (nM)	Fold increase of MIC_50_ value*
*L*. *plantarum* NCFB 965		
wild type	0.01 ± 0.003	1
Lp.JK-a	>1000	>100,000
Lp.JK-b	>1000	>100,000
Lp.JK-c	>1000	>100,000
Lp.JK-d	>1000	>100,000
*W*. *viridescens* NCFB 1655		
wild type	4 ± 1	1
Wv.JK-a	200 ± 100	50 ± 10
Wv.JK-b	140 ± 80	40 ± 10

*The relative MIC_50_ value presented indicates the fold increase in resistance against plantaricin JK of mutants derived from *L*. *plantarum* NCFB 965 and *W*. *viridescens* NCFB 1655 compared to the respective wild type strains.

### Identifying plantaricin JK-induced mutations by whole-genome sequencing

Based on previous results obtained with *W*. *viridescens* it was expected that genetic changes would be found in the same APC family protein gene as was identified by Oppegård et al. [20]. Indeed, the sequencing results confirmed that the *W*. *viridescens* mutants Wv.JK-a and Wv.JK-b had mutations in this gene (Table 2). In fact, the mutant Wv.JK-a protein had a point mutation at the same position (codon 377, amino acid Ala) as another mutant (JK2) previously identified by Oppegård et al. [20], introducing Val and Thr, respectively, in this position. The other mutant, Wv.JK-b, had a stop codon introduced at codon 191, leading to a truncated protein. Similarly truncated versions of the protein were produced by the frameshift mutation in codon 177 in three previously described mutants (JK4, 5 and 6) leading to termination in position 190 [20].

**Table 2:** Overview of mutations identified in mutants that are not present in the wild type strains of *L*. *plantarum* NCFB 965 and *W*. *viridescens* NCFB 1655.

Strains	Mutation	Contig	Position in contig
*L*. *plantarum* NCFB 965			
**Lp.JK-a**	ACA-ATA = T91I in cytosine permease protein	105	1480
	Insertion of 1047 nt after codon 270 in orf0070 APC family protein	13	55165
**Lp.JK-b**	Insertion of 14 nt in codon 98 in orf0070 APC family protein	13	54648
**Lp.JK-c**	CAA-TAA, Q83Stop in orf0070 APC family protein	13	54601
**Lp.JK-d**	Insertion of 1047 nt in codon 182 in orf0070 APC family protein	13	54900
*W*. *viridescens* NCFB 1655			
**Wv.JK-a**	GCG-GTG, A337V in APC family protein	10	3338
	ACG-GCG, T221A in FIG007079: UPF0348 protein family	10	11464
	CCG-CTG, P277L in dihydropteroate synthase	67	6845
	GCA-GCG, silent mutation in codon 52 in phosphopantetheine adenylyltransferase	82	2127
	TCT-CCT, S114P in dolichol-phosphate mannosyltransferase	101	3820
**Wv.JK-b**	Insertion of A, truncation after amino acid 190 (N191Stop) in APC family protein	10	2772
	ATG-ACG, M328T in phenylalanyl-tRNA synthetase beta chain	11	6392
	GAA-AAA, E39K in hypothetical protein CDS	18	2022

To further investigate the involvement of the APC family protein in resistance against plantaricin JK, we also analyzed plantaricin JK-resistance in *L*. *plantarum* by selecting and characterizing resistant mutants. The mutants, which all were fully resistant (>100,000 fold resistance) to plantaricin JK, carried mutations in an APC family protein 66% identical to the one identified in *W*. *viridescens*, further emphasizing that this protein is a probable plantaricin JK-receptor. In one of these mutants, Lp.JK-c, a stop codon had been introduced at codon 83. In the second mutant, Lp.JK-b, 14 nucleotides had been inserted in codon 98 leading to sequence alteration and truncation of protein 14 amino acids downstream of the insertion point. The two remaining mutants, Lp.JK-d and Lp.JK-a, contained a 1047 nucleotide transposon-like insert at codon 182 and 270, respectively, also leading to sequence alteration and truncation of protein after 5 and 19 amino acids post insertion, respectively. In addition, Lp.JK-a had a codon change from ACA to ATA in a cytosine permease gene leading to a Thr to Ile alteration in position 91 of the protein (Table 2). Three full-length copies of the transposon-like insert were present in the wild type *L*. *plantarum* genome (99.5% identical or more) and it is also present in a number of complete *Lactobacillus* genomes in GenBank (results not shown).

The mutants Wv.JK-a, Wv.JK-b and Lp.JK-a had additional mutations outside the APC transporter gene (Table 2). The possible contribution of these additional mutations to the plantaricin JK-resistance was not pursued further.

### Correlating mutations with a potential docking site

In the previous study by Oppegård et al. [20], all mutations resulting in the substitution of a single amino acid were at positions in or near a predicted membrane pore-lining helix (TM helix 10) or in an extracellular loop between TM helix 11 and 12. This was also the case in this study. Of the six plantaricin JK-resistant mutants characterized (Table 1), one (Wv.JK-a) had a mutation resulting in the substitution of a single amino acid in TM helix 10, i.e. Ala in position 377 to Val. The other five mutants, denoted Wv.JK-b from *W*. *viridescens* and Lp.JKa-d from *L*. *plantarum*, all contained mutations giving rise to truncated versions of the protein, either due to premature stop codons or to insertions. Interestingly, the *L*. *plantarum* NCFB 965 truncating mutations led to full resistance against the bacteriocin (over 100,000 times more resistant than the wild type strain, see Table 1). This is in contrast to similar truncations in *W*. *viridescens*, both from this study (Wv.JK-b) and the previous study [20], that led to 50–600 times increase in resistance. This suggests that other, less efficient plantaricin JK-receptors, possibly also APC family proteins, may be present in *W*. *viridescens* NCFB 1655, but not in *L*. *plantarum* NCFB 965.

One could hypothesize that plantaricin JK inserts in the membrane and then interacts with the TM helix 10 as well as the extracellular loop between TM helix 11 and 12. These two regions would then together function as a docking site for plantaricin JK. If these regions are important for interaction between the receptor and plantaricin JK, one would expect the regions to be conserved in bacterial strains that are sensitive to plantaricin JK. A comparison of *W*. *viridescens* NCFB 1655 and *L*. *plantarum* NCFB 965 regarding the amino acids comprising TM helix 10 and the extracellular loop between TM helix 11 and 12 reveals that the sequences are, respectively, approximately 88 and 60 percent identical in these two regions (compared to an overall identity of 66% for the complete sequences; results not shown). Conserved amino acids comprising TM helix 10 includes an Ala residue at position 377 where a mutation has been observed twice, once in this study and once in our previous study [20], as well as S387P (JK1) and W395Stop (JK10; also from the previous study by Oppegård et al. [20]). Conserved residues in the extracellular loop between TM helix 11 and 12 include a His residue at position 428 where two previously characterized mutants (JK8 and JK9) contained an Arg (H428R) [20]. Interestingly, the amino acid at position 425 is not identical in the two organisms (M425 in *W*. *viridescens* and F425 in *L*. *plantarum*), although the M425K-mutation in *W*. *viridescens* (JK11) resulted in 400-fold increase in resistance (Oppegård et al. [20]). This might imply that a positively charged residue, such as Arg and Lys, at this position leads to electrostatic repulsion between the net positively charged plantaricin JK and the potential target site. This may also be the case for the mutants JK8 and JK9 mentioned above in which His is mutated to Arg (H428R) [20].

**Fig 1:**
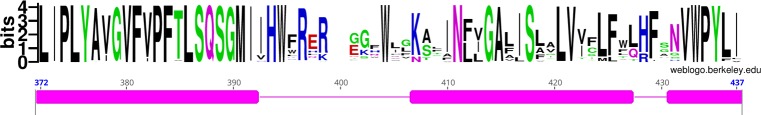
A consensus sequence illustrating the conserved residues in the regions of interest in the membrane protein. The consensus sequence using WebLogo (weblogo.berkeley.edu) was generated through a search with protein-BLAST using the APC family protein from *W*. *viridescens* NCFB 1655 as a template. The alignments reveal a highly conserved region spanning almost the entire TM helix 10 and to a lesser extent the loop between TM helix 11 and 12. The predicted TM helices 10–12 (from left to right) are indicated below as purple bars.

To further investigate whether the identical amino acids in these two regions of *W*. *viridescens* and *L*. *plantarum* also are conserved among other related strains, a search using Basic Local Alignment Search Tool (BLAST) with the relevant part of the APC family protein from *W*. *viridescens* as query was performed. The 200 closest related GenBank protein sequences were aligned using Clustal Omega [26], and a sequence logo was constructed from the alignment using WebLogo (weblogo.berkeley.edu). As the logo in Fig 1 shows, TM helix 10 is almost fully conserved in these 200 sequences, in contrast to the remaining part of this region. Interestingly, Arg is commonly observed as an alternative to His in the extracellular loop between TM helix 11 and 12 at position 428 where a His to Arg substitution led to increased plantaricin JK resistance in the previously described mutants JK8 and JK9. This may suggest that the His to Arg mutation disturbs the interaction between the bacteriocin and the protein without interfering with the function of the protein. It also implies that His in this position does not by itself lead to plantaricin JK sensitivity, since it is unlikely that the hosts of all His-containing proteins in the alignment are sensitive.

Apart from a His or Arg residue at position 428 and a Phe residue at position 429, none of the residues in the loop between TM helix 11 and 12 seem to be highly conserved. In contrast, out of the 24 residues comprising TM helix 10, all but three of them are identical (Fig 1). This indicates that the residues in this presumably pore-lining helix are important for the primary function of the APC family protein.

In an attempt to visualize the regions of interest, a protein structure model was constructed using I-TASSER [27–30] with the protein sequence from *W*. *viridescens* NCFB 1655 as input. The highest ranked 3D model (Model 1) by I-TASSER was constructed using another APC superfamily protein, ApcT from *Methanocaldococcus jannaschi* [31] (PDB-ID: 3gi9), with 17% sequence identity and 70% query sequence coverage, as template. Model 1 had the following parameters listed: confidence score (C-score) of—1.08, template modeling score (TM-score) of 0.58 ± 0.14 and a root mean square distance (RMSD) value of 10.3 ± 4.6Å. C-score is a confidence score used for evaluating the quality of predicted models. C-score is normally in the range [–5, 2] where a higher score generally means a model with high confidence. A TM-score illustrates the global fold similarities between two structures and is independent of the protein length. A TM-score >0.5 is indicative of a model with correct topology whereas a TM-score <0.17 means random similarities [30]. The high C- and TM-scores indicate that Model 1 provides a good implication of the secondary and tertiary structure of the protein. In contrast to the TM-score, which weights pairs of residues with small distances higher than residues with larger distances, RMSD averages the distance of pairs of residues between two structures with an equal weight and is thus sensitive to local structural differences, such as loops and unstructured tails [30].

We also modeled the *W*. *viridescens* protein using Phyre2 [32] and obtained a 3D structure with a similar topology as Model 1 obtained by I-TASSER, and with a confidence score of 100% with the first four templates identified. A Phyre2-denoted confidence score measures the likelihood of homology and a score >90% generally implies that the query protein adopts the overall same fold as the template [32]. Using MOLMOL we compared the membrane-associated regions of the two predicted structures and calculated the backbone RMSD value to be 2.68Å, indicating that the positions of the alpha carbon atoms are quite similar in the two models. The high degree of similarity of the two 3D models of the APC family protein obtained from two different structure prediction servers implies that the topology and general packing/folding of the 12 TM helices in the two models are close to the true structure of this region of the protein.

**Fig 2:**
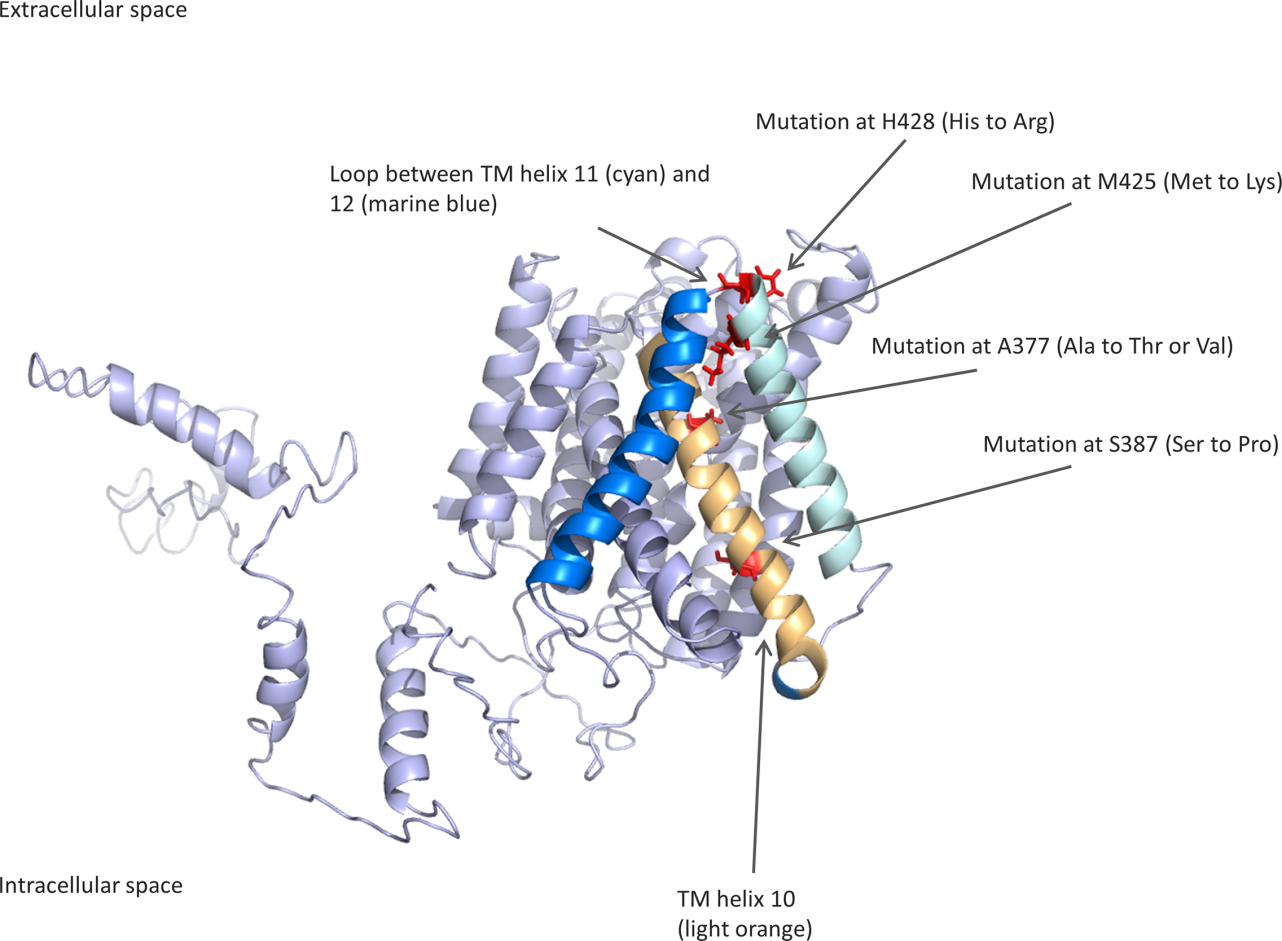
Side view of the I-TASSER-predicted structure of the APC family protein of *W*. *viridescens* NCFB 1655. The mutated amino acids are highlighted in red. The TM helix 10 is colored in light orange, TM helix 11 in cyan and TM helix 12 in marine blue. The structure is based on a known crystal structure from another APC superfamily member [31].

**Fig 3:**
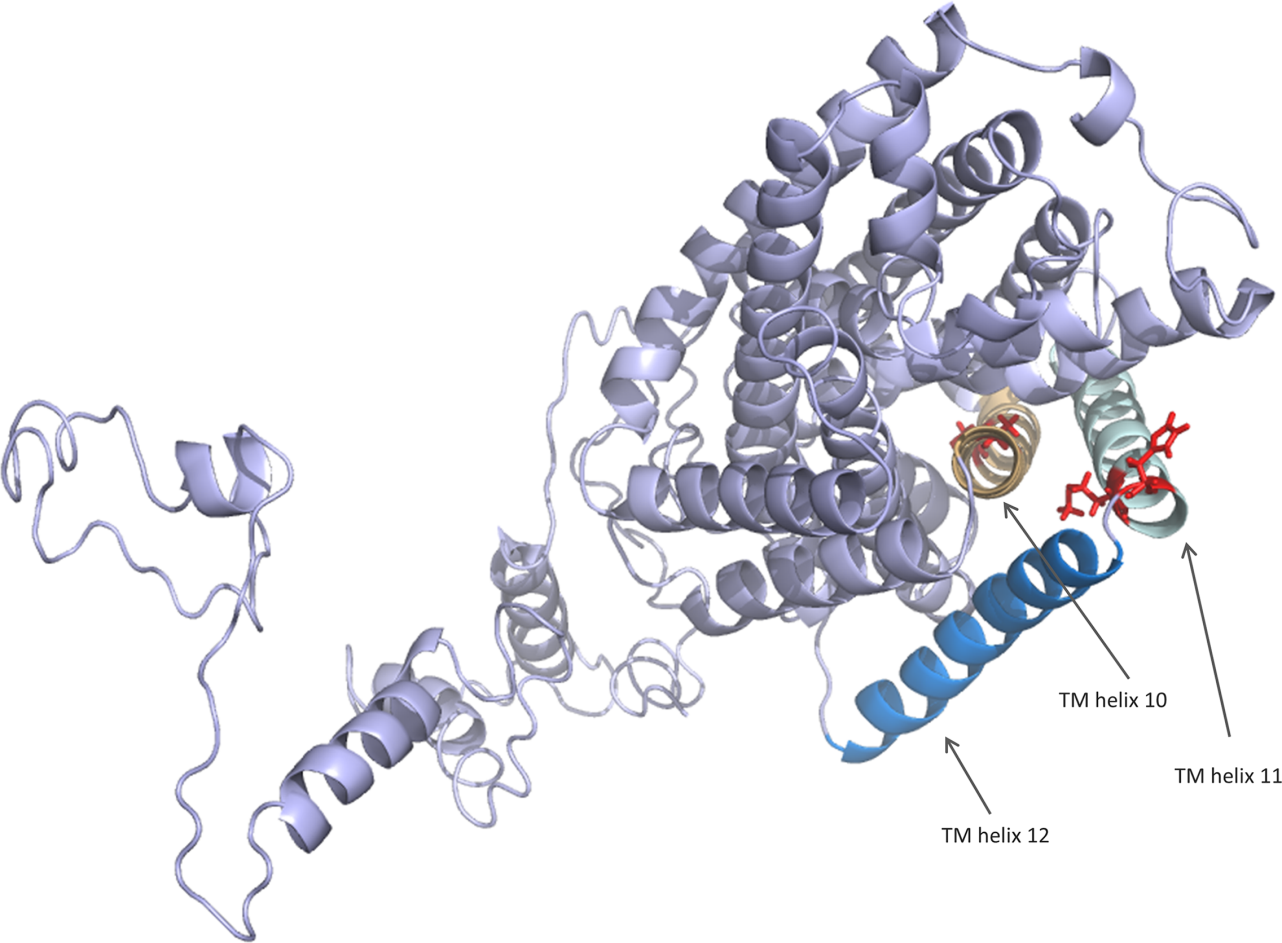
Top view of the I-TASSER-predicted structure of the APC family protein of *W*. *viridescens* NCFB 1655. The predicted structure illustrates that TM helix 10 is part of a membrane pore and that the loop between TM helix 11 and 12 are close to TM helix 10 in space. See Fig 2 for color-codes.

Figs 2 and 3 show the structure of Model 1 obtained by I-TASSER. The predicted 3D structure of the *W*. *viridescens* protein is in concordance with the predicted secondary structure of the protein (Fig 2 in the present study and Oppegård et al. [20]), both with regard to the localization of transmembrane regions and the intra- and extra-cellular regions. The position of helix 10 in the structure also fits with the prediction that it is a pore-lining helix (Oppegård et al. [20]). This again suggests that the general features of the predicted structure are correct.

In the predicted structure, the mutations at position 425 and 428 are located at or near the extracellular loop between TM helix 11 and 12. Furthermore, the mutations located at 377 and 387 are positioned in the middle or near the upper part of TM helix 10. Fig 3 indicates that TM helix 10 is part of an inner membrane pore and that the extracellular loop between TM helix 11 and 12 are in close proximity to TM helix 10. Tentatively, this structure reveals a potential docking site for plantaricin JK at these regions, suggesting that these mutations might lead to decreased interaction with the bacteriocin due to distortion in 3D structure (e.g. S387P) or due to less contact points between the protein and bacteriocin (e.g. A377V and A377T) or even repulsion between the two (e.g. M425K and H428R).

The identification and characterization of bacteriocin receptors and the bacteriocin docking site on these receptors is important for gaining detailed insight into the mode of action of bacteriocins and their development into therapeutic drugs and/or natural additives in food. The method used in this study, possibly in combination with molecular dynamic simulations, should be an applicable and general approach for identification of bacteriocin receptors and docking sites.
